# Genome-wide association studies for inflorescence type and remontancy in *Hydrangea macrophylla*

**DOI:** 10.1038/s41438-020-0255-y

**Published:** 2020-03-01

**Authors:** Xingbo Wu, Lisa W. Alexander

**Affiliations:** 1 Oak Ridge Institute of Science and Technology, Otis L. Floyd Nursery Research Center, 472 Cadillac Lane, McMinnville, TN USA; 2 U.S. Department of Agriculture, Agricultural Research Service, U.S. National Arboretum, Floral and Nursery Plants Research Unit, Otis L. Floyd Nursery Research Center, 472 Cadillac Lane, McMinnville, TN USA

**Keywords:** Genetic markers, Plant breeding

## Abstract

Inflorescence type and remontancy are two valuable traits in bigleaf hydrangea (*Hydrangea macrophylla* L.) and both are recessively inherited. Molecular marker-assisted selection (MAS) can greatly reduce the time necessary to breed cultivars with desired traits. In this study, a genome-wide association study (GWAS) using 5803 single-nucleotide polymorphisms (SNPs) was performed using a panel of 82 bigleaf hydrangea cultivars. One SNP locus (*Hy_CAPS_Inflo*) associated with inflorescence type was identified with general linear model (GLM) and mixed linear model (MLM) methods that explained 65.5% and 36.1% of the phenotypic variations, respectively. Twenty-three SNPs associated with remontancy were detected in GLM whereas no SNP was detected in MLM. The SNP locus (*Hy_CAPS_Inflo*) was converted to a cleaved amplified polymorphic sequence (CAPS) marker that showed absolute identification accuracy (100%) of inflorescence type in a validation panel consisting of eighteen *H. macrophylla* cultivars. The SNP was investigated in 341 F_1_ progenies using genotyping by sequencing (GBS) and co-segregated with inflorescence type (*χ*^2^ = 0.12; *P* = 0.73). The SNP was subsequently used for breeding selection using kompetitive allele specific PCR (KASP) technology. Future directions for the use of genomics and MAS in hydrangea breeding improvement are discussed. The results presented in this study provide insights for further research on understanding genetic mechanisms behind inflorescence type and remontancy in *H. macrophylla*. The CAPS and KASP markers developed here will be immediately useful for applying MAS to accelerate breeding improvement in hydrangea.

## Introduction

*Hydrangea macrophylla* L. is a popular cultivated horticultural crop widely grown throughout America, Asia, and Europe^1,2^. Famous for its appealing large corymbs and unique flower color changes, hundreds of named cultivars has been developed and selected throughout the world^3^. Hydrangea is a versatile ornamental plant that can be used as a florist, potted, and landscape plant. Breeding programs in hydrangea usually have specific goals depending on how the plants will be used. Breeding landscape hydrangeas focuses on whether the plant forms a rounded or mounded shrub composed of erect, unbranched stems, whereas florist hydrangea breeding programs target stem strength and durability^4^. However, flowering traits are always considered as a priority for any hydrangea breeding program, as flowers present the key horticultural attraction for hydrangea as for many other ornamental crops. Sepal coloration, inflorescence type, and the timing of flower bud initiation are the most important flowering traits in hydrangea. While flower color changes in hydrangea have been shown to be a result of internal detoxification of Al under low-pH conditions^5^, inflorescence type and the timing of flower bud initiation are largely under genetic control^6–8^.

Inflorescence type is the most obvious horticultural trait in *H. macrophylla* cultivars. Two types of inflorescence, mophead, and lacecap, are categorized by the relationship between the small, fertile flowers and the large, showy sepals. The showy sepals of mophead hydrangeas completely surround the fertile flowers located on the pedicels below, leading to a rounded inflorescence. Lacecap inflorescences consist of a plane of fertile flowers surrounded by a ring of showy sepals leading to a flat inflorescence^9^. “Endless Summer” The Original (“Bailmer”) and “Veitchii” are two *H. macrophylla* cultivars that represent the different inflorescence types. “Endless Summer” is a pink- or blue-flowered (pH dependent) hydrangea with mophead inflorescences varying from 7.5 to 15 cm in diameter^10^, while “Veitchii” has lacecap flower heads with large white sepals. Breeding efforts have been primarily focused on producing cultivars with mophead inflorescences given customer preference.

Remontancy, or reblooming, is the ability to initiate floral buds on new vegetative growth which leads to continuous flowering throughout the growing season. Remontancy is a highly desired trait for modern landscape plants, as consumers consistently choose varieties with season-long floral display and reliable blooming in more northerly or frost-prone areas^11^. Well-known remontant *H. macrophylla* cultivars, include “All Summer Beauty”, “Bailmer” (marketed as “Endless Summer”^®^), “David Ramsey”, “Decatur Blue” “Early Sensation” (marketed as “Forever and Ever”^®^), “Oakhill”, and “Penny Mac”^6,7,9^. New remontant cultivars are often introduced as collections within an established brand, such as the Endless Summer^®^ series, Forever & Ever^®^ series, and Let’s Dance^®^ series. So Long^®^ is the latest series of reblooming hydrangeas launched in 2017 by SAPHO (Syndicate for the improvement of ornamental horticultural plants), the exclusive licensing partner of INRA.

Breeding procedures for horticultural crops based on 4–6 years of field evaluation are costly and inefficient given that the majority of plants will lack desired trait combinations. Trait-associated molecular markers can be used to select desired plants at their seedling stage to shorten the breeding time and increase the speed of cultivar development. Single-nucleotide polymorphisms (SNPs) are third-generation DNA molecular marker technology defined as single-base changes at a specific nucleotide position across the entire genome of all organisms^12^. Compared to traditional molecular markers, SNPs are abundant, stable, and easily detectable by sequencing technologies such as genotyping-by-sequencing (GBS) even without a priori genome sequence information^13^. Thus, SNP markers have been widely used in genetic diversity assessments, molecular evolution studies, and genetic mapping for traits of interest in diverse horticultural crops such as carnation^14^, chrysanthemums^15^, roses^16^, lilies^17^, and tulips^18^.

Genome-wide association study (GWAS) is a powerful tool for dissecting complex traits in plants by correlating large numbers of molecular markers distributed across the genome with phenotypic variation^19^. In the past few years, GWAS has been applied in many plant species to find functional alleles that can assist breeding programs^20^. In roses, key genes that responsible for prickle density and the number of flower petals has been found by using GWAS^21^. A key gene that controlling fruit shape was identified by exploiting natural variation in a panel of peach accessions^22^. Significant SNPs identified from GWAS can be converted into readily usable molecular markers, such as single-strand conformation polymorphisms^23^, high resolution melting^24^, kompetitive allele-specific polymerase chain reaction (PCR) (KASP)^25^, and cleaved amplified polymorphic sequences (CAPSs)^26^ or derived CAPSs (dCAPSs)^27^. Among them, CAPS is the most simple, efficient and economic technique for in-lab SNP detection, especially for frequent and small quantity breeding selection, whereas KASP is more suitable for a large population selection program.

Conventional breeding of woody ornamental nursery crops can be improved greatly by using molecular markers associated with traits of interest. Accelerated breeding of new *H.* *macrophylla* cultivars directly helps the nursery industry because consumer interest is driven in part by the release of new, novel plants. To this end, we developed 5803 SNPs for a cultivated *H. macrophylla* panel using GBS technology. The goal of this research was to (1) identify SNPs associated with inflorescence type and remontancy in *H. macrophylla* via GWAS, and (2) convert trait-associated SNPs into lab-friendly molecular markers for MAS of hydrangea.

## Material and methods

### Plant material and genotyping

A total of 82 *H. macrophylla* cultivars were included in this study (Table S1). Hydrangea plants were collected from public or commercial sources and maintained in 5 or 7-gallon containers at the Otis L. Floyd Nursery Research Center in McMinnville, TN. Fresh leaf tissue was collected directly from plants into a 2 ml Eppendorf tube with lysis buffer and ground in a FastPrep-24^™^ 5G homogenizer. Genomic DNA was isolated from the lysed tissue using DNeasy Plant Mini Kit (Qiagen) followed by RNase treatment. Nucleic acid was evaluated in 1% agarose gel and quantified using a spectrometer (NanoDrop 2000, Thermo Scientific, Wilmington, DE). Genotyping of the hydrangea cultivar panel was carried out via GBS as described by Elshire et al.^28^. A total of 5803 high-quality SNPs discovered and described previously^29^ were used to perform genome-wide association analysis in the present study.

### Genetic and population structure analysis

Population structure was investigated using STRUCTURE 2.3.0 software with admixture mode^30^. The number of subpopulations, K, was set from 1 to 10 with 100,000 burn-in and 100,000 Markov Chain Monte Carlo with 10 times of iteration. The output of STRUCTURE was evaluated in STRUCTURE HARVESTER^31^ to determine the best *K* value based on Evanno test^32^. Phylogenetic study was carried out using neighbor-joining method in MEGA 7^33^, with 500 bootstraps to estimate nodal probabilities.

### Identification of SNP associated with inflorescence type and remontancy

**Fig. 1:**
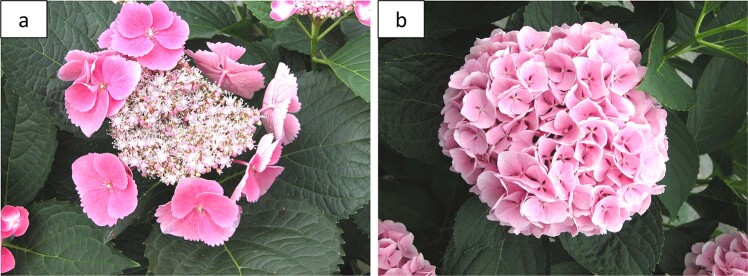
Two inflorescence types of Hydrangea macrophylla. **a** Lacecap inflorescence; **b** mophead inflorescence. Photos: L. Alexander, 09 June 2016, McMinnville, TN

The characterization of hydrangea inflorescence type was based on a previous study published by Reed et al.^9^ with multiyear observations. Flower inflorescence type was considered as a qualitative trait and categorized as lacecap (L) or mophead (M; Fig. 1). Only cultivars with published evidence of remontancy were considered remontant; free-flowering cultivars or those with anecdotal remontancy were not considered remontant (Table S1). Remontancy was considered as a qualitative trait and categorized as remontant (YES) or nonremontant (NO).

Based on the filtered SNP data, the GWAS analysis was conducted using TASSEL v5.0 software with two models: general linear model (GLM) and mixed linear model (MLM) + Q, a MLM considering population structure (Q) and kinship (K) as covariates. The significance threshold was set to *P* ≤ 0.001 for marker–trait associations and then adjusted using the Bonferroni threshold (*P* ≤ 0.05/5803 = 8.7E^−6^) to reduce false-positive associations. The proportion of the phenotypic variation explained (PVE) by each marker was estimated by the relevant *R*^2^. SNPs that were repeatedly detected by all the models were considered as high-confidence GWAS results.

### Development and verification of SNP marker using CAPS and GBS approach

The SNPs that were suitable for conversion to CAPS markers and the corresponding restriction enzymes were selected using dCAPS Finder 2.027^34^. The specific primers were designed using DNASTAR^35^. Primer sequences are as follows: GCTACAGCATACTGATTATCTCC (forward) and TGGAGGTCTTAATGCTCATAGAA (reverse). Eighteen *H. macrophylla* cultivars or breeding accessions with determined inflorescence type were used to verify the CAPS markers (Table S2). The genomic DNA of the above materials was extracted from young leaves using DNeasy Plant Mini Kit (Qiagen). The DNA was diluted to a final concentration of 50 ng/μL for PCR verification. The PCR reactions were conducted in a total volume of 25 μL, including 12.5 μL GoTaq Colorless Master Mix (Promega), 1 μL of each primer (10 μm/μl), 2 μL of template DNA, and 8.5 μL of ddH_2_O. The PCR protocol consisted of an initial denaturation at 94 °C/3 min followed by 35 cycles of 94 °C/30 s, 57 °C/30 s, and 72 °C/30 s and finally an elongation step of 72 °C/7 min. The amplified PCR products were sequenced to confirm the SNP site and digested by restriction endonuclease *Bmg*BI according to the manufacturer’s instructions (New England Biolabs, NEB, USA). The digestion products were separated via 2% agarose gel.

In addition, a total of 341 F_1_ progenies from a cross between the lacecap cultivar “Veitchii” and mophead cultivar “Endless Summer” were used to verify the phenotype–genotype association. Genomic DNA was prepared and submitted for GBS following the same protocol as stated above. SNPs were called via the TASSEL UNEAK pipeline using the same parameters described by Wu and Alexander^29^. Inflorescence type (mophead or lacecap) was investigated for genotype–phenotype association using chi-squared analysis.

### Application of SNPs in MAS

Large-scale MAS requires the application of a high-throughput genotyping system to minimize cost and maximize efficiency. For this purpose, a KASP-SNP markers were also developed for MAS. A recently developed F_2_ population [(“Veitchii” × “Endless Summer”) × (“Veitchii” × “Endless Summer”)] resulting from two heterozygous progenies of the F_1_ cross mentioned above was tested for selection purposes. The DNAeasy Plant Mini (Qiagen) extraction kit was used for genomic DNA isolation in F_2_ progenies according to the manufacturer’s kit instructions. The concentration and quality of the DNA samples was determined in NanoDrop2000 spectrophotometer (Thermo Fisher Scientific) and checked in 1% agarose gel. DNA samples were then diluted to the appropriate level for SNP genotyping (10 ng mL^−1^) as required.

Sequences that were 100 bp long on each side of the SNP were submitted for primer design. KASP genotyping assay includes KASP master mix containing the common fluorescent reporting dyes FAM and HEX, along with Rhodamine X (ROX) as a passive reference background dye. The KASP assay mix and primers were developed and run by LGC Biosearch Technologies (Beverly, MA) using the KASP on demand platform^36^. SNP alleles were determined with KlusterCaller software^37^.

## Results

### Genetic diversity and population structure

**Fig. 2:**
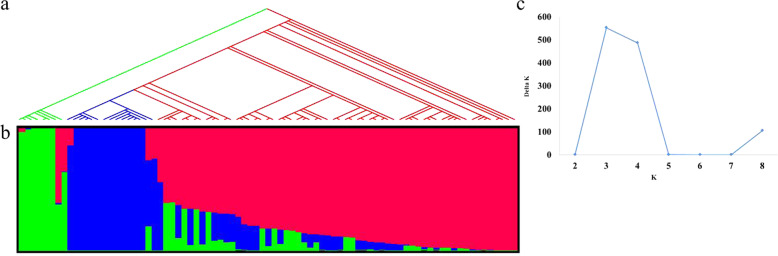
Population structure analysis of a Hydrangea macrophylla cultivar panel based on 5803 SNPs developed by GBS. Each individual is represented by a vertical bar, reflecting assignment probabilities to each of the three groups. Group I: green bars; group II: blue bars; group III: red bars. **a** Phylogenetic tree based on the neighbor-joining method in MEGA 7; **b** plot of Δ*K* value with *K* from 2 to 10 based on Evanno test; **c** population structure based on mixed-model analysis using STRUCTURE software. Phylogenetic studies corresponded to population structure groups. ssp. *serrata* (coded as green) agreed with group I, ssp. *macrophylla* separated into two clades (colored as blue and red) and corresponded to group II and group III, respectively

A neighbor-joining phylogenetic tree based on 5803 discovered SNPs classified the 82 cultivated hydrangeas into two major clades according to their subspecies category (Fig. 2a). Six cultivars from subspecies *serrata* were clustered as clade I (represented by green). Cultivars from subspecies *macrophylla* formed into two subclades named as clade II and III (represented by blue and red, respectively). Structure analysis identified three populations in the cultivar panel based on Evanno test (Fig. 2b). The three groups (Groups I–III) consisted of 6, 15, and 35 cultivars, respectively (Fig. 2c). Group I included all six cultivars from subspecies *serrata*, corresponding to the clade I. Group II and Group III consisted of all cultivars from subspecies *macrophylla*, corresponding to the clade II and clade III. Of which, 15 cultivars that consisted of Group II correspond to clade II and 35 cultivars from the rest of *macrophylla* subspecies that was in clade III fall into Group III.

### GWAS of inflorescence type and remontancy

**Fig. 3:**
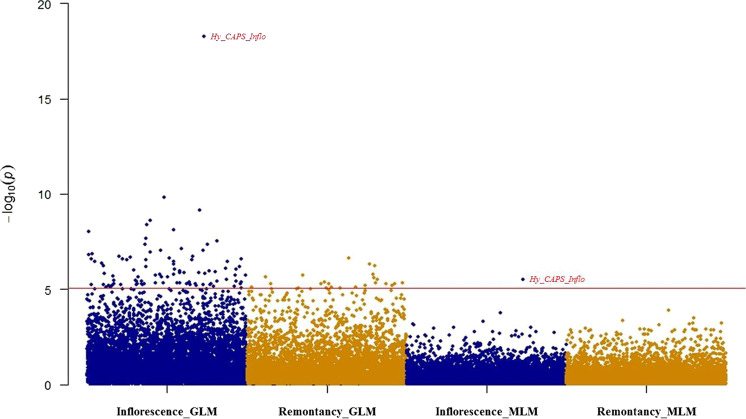
Manhattan plot of inflorescence type and remontancy in two simulation models (GLM and MLM) resulting from GBS-GWAS with 5803 SNPs in *Hydrangea macrophylla*

Out of 82 hydrangea cultivars, 29 showed mophead inflorescences and 53 showed lacecap inflorescences. A total of 94 SNPs were significantly associated with inflorescence type in the GLM model, and they explained 22.3–65.5% of the variation in hydrangea inflorescence type in this panel. However in the MLM-Q model, only one SNP was found to be significantly associated with inflorescence type, explaining 36.12% of total phenotypic variation. One SNP possessing C/T polymorphism was found to be the shared SNP. This SNP also showed the highest PVE in both models (Fig. 3).

Thirteen cultivars were classified as remontant based on published information (Table S1). A total of 23 SNPs were detected using GLM, explaining 23.1–33.7% of the variation in remontancy. No SNP was detected in MLM + Q for remontancy under the designated threshold (Fig. 3).

### Development and verification of SNP marker

**Fig. 4:**
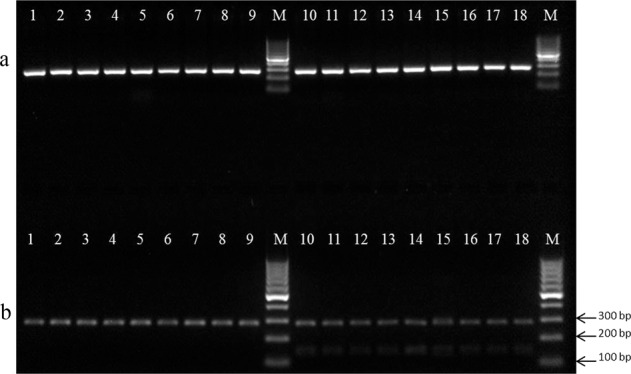
Agarose gel electrophoresis for undigested (**a**) and digested (**b**) CAPS marker (*Hy_CAPS_Inflo*) products. Lanes 1–9 represent 9 mophead *H. macrophylla* cultivars, and lanes 10–18 represent 9 lacecap *H. macrophylla* cultivars. Lane 1 (“Endless Summer”) and Lane 10 (“Veitchii”) were the parents of the F_1_ progenies in Lane 17 (0872-053) and Lane 18 (0872-076) that were used as the parents of the F_2_ population

One SNP that explained the highest PVE for inflorescence type in two models was selected for marker development. To develop specific CAPS markers, we introduced a 0 mismatch into the forward or reverse primer, and selected common restriction enzymes as potential candidates to design the primers in this study. As a result, a CAPS marker based on the restriction enzyme *Bmg*BI was developed (termed *Hy_CAPS_Inflo*). Eighteen genotypes were included in the validation panel. A single fragment of 278 bp was obtained from the PCR product across the validation panel, indicating specific and stable PCR amplification. Two types of band combinations were observed in the digested PCR product. In lacecap hydrangeas, PCR fragments were digested into two bands of 148 and 130 bp (undetectable due to 21 bp difference), along with the undigested 278 bp PCR product. The PCR product was not digested in mophead hydrangeas, showing only one band of 278 bp. Two well-known hydrangeas “Endless Summer” (mophead, Lane 9) and “Veitchii” (lacecap, Lane 19) showed different band types as expected. The CAPS marker derived from the SNP exhibited 100% efficiency in identifying mophead and lacecap inflorescence types in the first validation panel consisting of eighteen *H*. *macrophylla* cultivars and breeding accessions (Fig. 4; Table S2). The developed CAPS marker co-segregated with inflorescence type in hydrangea for all samples. Sequencing results of the PCR products confirmed that “Endless Summer” is homozygous with the T/T allele and “Veitchii” is heterozygous with the C/T allele, with their two F_1_ progenies “0872-053” and “0872-076” being heterozygous.

Out of 341 F_1_ progeny, 316 were used to compare inflorescence phenotype with genotype. In total, 116 (36.71%) had mophead flowers and T/T genotype, 181 (57.28%) had lacecap flowers and C/T genotype (Table 1). Eleven progenies showed mismatch between their phenotype and genotype: 7 lacecap progenies were T/T genotype and 4 mophead progenies were C/T genotype. In addition, 8 lacecap progenies were C/C genotype. Categorizing these plants by inflorescence type, they segregated into 120 (38.0%) mophead plants and 196 (62.0%) lacecap plants. The SNP called from the F_1_ population using GBS showed 98.5% efficiency of identifying inflorescence type. Chi-squared analysis for segregation of inflorescence type did not support the hypothesis that inflorescence type is controlled by a single recessive gene; however, the chi-square value for phenotype–genotype association indicated that SNP marker *Hy_CAPS_Inflo* co-segregated with inflorescence type in *H. macrophylla* (Table 1).

**Table 1:** Segregation of lacecap and mophead inflorescence type in 316 F1 progenies derived from *Hydrangea macrophylla* “Veitchii” × *H*. *macrophylla* “Endless Summer” and their corresponding SNP genotypes

Phenotype	Genotype			Total	*χ*^2^ value	*P*
	C:C	C:T	T:T			
Lacecap	8	181	7	196	18.28	0.000^a^
Mophead	0	4	116	120		
Total	193		123		0.12	0.728^b^

^a^Test for phenotype segregation (1:1) of inflorescence type

^b^Test for phenotype–genotype association of inflorescence type with the SNP locus

### Marker-assisted selection using KASP genotyping

**Fig. 5:**
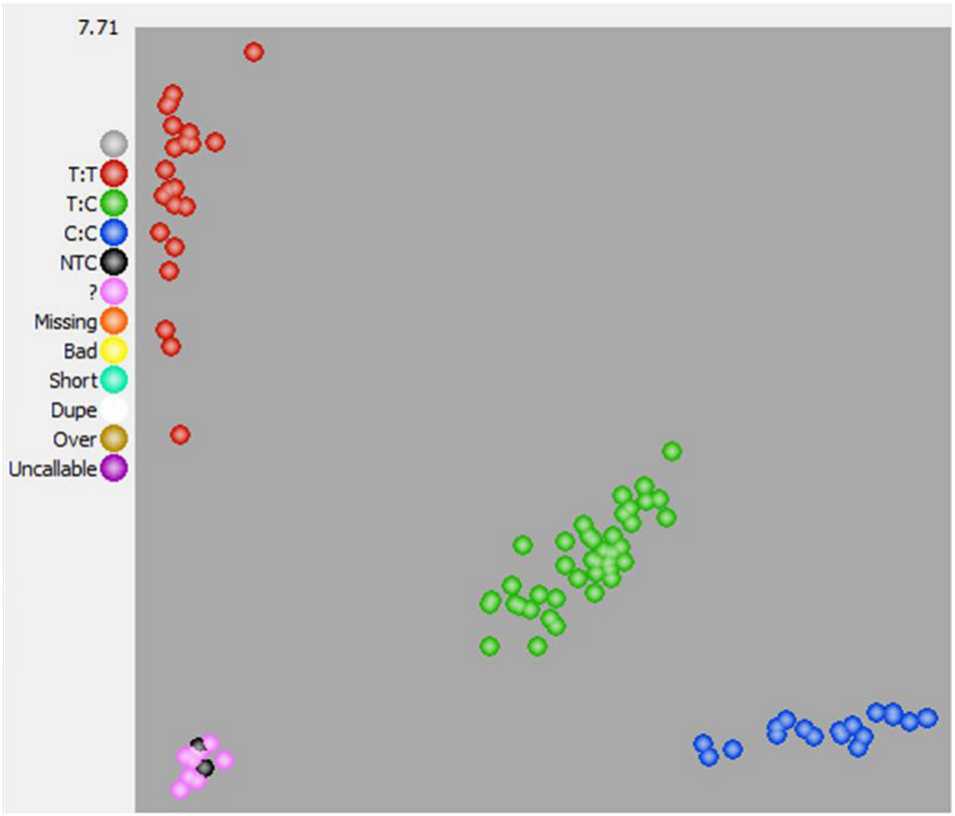
Genotype plot for *Hy_CAPS_Inflo* using the KASP on demand (KOD) platform for 73 F_2_ progenies from a bi-parental breeding population, the two F_1_ parents, and a no-template control. Three genotypes (T:T, C:T, and C:C) were observed in 17, 39, and 14 progenies

A recently developed F_2_ population [(“Veitchii” × “Endless Summer”) × (“Veitchii” × “Endless Summer”)] was used to investigate the segregation of the discovered SNP locus as well as for trait selection. Out of 73 F_2_ progenies that were tested for inflorescence type by the KASP-SNP marker, 70 were successfully genotyped resulting in a 95.9% calling rate (Fig. 5). Three progenies did not have a successful allele calling and were removed from further analysis. Seventeen progenies were homozygous recessive representing the T/T genotype (red), thirty-nine were heterozygous representing the C/T genotype (green), and fourteen were homozygous dominant representing the C/C genotype (blue). The SNP locus segregated according to the expected 1:2:1 ratio. Three-fourths of the progenies were detected to be lacecap genotype.

## Discussion

*H. macrophylla* is a species with two ploidy levels, diploid and triploid^38^. The diploid genome size is about 2.0 Gb with high levels of heterozygosity^39^. A lack of genomic information hampers the performance of hydrangea molecular genetic analyses and breeding progress, particularly of exploring trait-related molecular markers. To date, SSR markers are still the most popular molecular marker type in hydrangea breeding programs, but their usefulness remains limited by low marker numbers^40^. Recently, a large number of SNPs were developed using GBS technology for ongoing genomic and breeding applications in hydrangea^29^.

GWAS is a powerful tool for identifying the genetic loci and candidate genes responsible for the natural variation in horticultural traits. GWAS often requires high marker density, large population sizes, and appropriate statistical models^41^. However, the performance of GWAS also depends on many factors such as plant species, analysis platform, and the genetic nature of target traits^15^. GWAS performed in 1580 peach accessions failed to determine candidate genes that associated with qualitative traits but succeeded in a later study with 129 peach accessions^22^. A major SNP locus was associated with waterlogging tolerance in a panel of 88 chrysanthemum accessions and was used for further breeding selection^15^. GWAS have also been successfully conducted in canola^41^ and rose^42^ using small populations containing less than 100 sample entries. Here, GWAS was performed in 82 hydrangea accessions and a major SNP locus was identified to be associated with inflorescence type, which was known to be under single-gene control^8^. The success of the current study further demonstrates the possibility of performing GWAS in a relatively small panel under certain conditions such as sufficient marker density and prior information of the genetic nature of targeted traits.

The statistical model is also a key component that affects the power of GWAS. In the GLM, 94 SNP loci were identified to be associated with inflorescence type with the PVE ranging from 22.27 to 65.51%. Compared to GLM, the MLM statistical model allows for a large reduction in spurious associated SNPs with only one SNP being identified with 36.12% PVE. Twenty-three SNP loci were identified in the GLM but none was detected in MLM model. As expected, the MLM was more stringent than the GLM as reported in many other ornamental crops^15,22,43^. Among these SNPs, only the leading SNP associated with inflorescence type was found in both models. Given the genetic nature of this particular trait, the associated SNP locus was converted to PCR-based markers for further validation as well as molecular marker selection in hydrangea breeding.

The validation of the SNP marker using PCR-based and GBS methods exhibited 100% identification efficiency in the validation panel and 98.47% identification efficiency in the F_1_ population, respectively (Fig. 4, Table 1). There are several possible explanations for the mismatch of genotype and phenotype in the eleven F_1_ progenies. In regards to the genotyping aspect, GBS is a skim-sequencing technology aiming at genome-wide SNP discovery through bioinformatics tools. Errors could occur in both sequencing and SNP calling procedures. In wheat, four SNPs discovered by GBS during preharvest sprouting QTL mapping did not show the same results when validated by KASP-SNP genotyping, showing instead a 1.35% sequencing or SNP determination error in GBS^44^. There was also possible phenotyping error in determining the inflorescence type. The key determination of mophead and lacecap hydrangea is the ratio and placement of showy sepals and non-decorative flowers, but it is not obviously distinguishable in all cases. For example, the *H. macrophylla* cultivar Uzu Azisai (aka Ayesha) produces mophead inflorescences often confused as lacecap due to the many, small flowers and the absence of large, showy sepals. Also, the F1 population may have contained triploids due to unreduced gamete formation in the parents^45^. The presence of triploids in an F1 population has been linked to skewed segregation ratios^46^. Finally, the mismatch of genotype and phenotype could be due to the basic methodology of GBS. GBS is a reduced representation genotyping method that does not produce a whole-genome sequence^28^. Even if the significant SNP is tightly linked to the causative gene, there still exists the possibility that recombination between the gene and the marker could occur.

Even though the segregation in the F_1_ population did not support a 1:1 ratio between mophead and lacecap inflorescence types, the chi-square test did indicate that SNP marker *Hy_CAPS_Inflo* co-segregated with inflorescence type in the F_1_ population. However, the heredity of this particular SNP did fit the 1:2:1 ratio in a recently developed F_2_ population derived from two heterozygous F_1_ progenies (Fig. 5). The reason for the skewed segregation in the F_1_ population is not clear. Segregation distortion has been observed in many other plants but the underlying mechanism is not fully understood^47^. Chromosomal translocation, competition among gametes, and the inheritance of alleles affecting the viability of the zygote, embryo, or seedling are all possible reasons for segregation distortion, but intentional or unintentional selections are the most significant factor in breeding programs^48,49^.

Mophead inflorescence was reported to be a recessive characteristic controlled by a single recessive gene located on linkage group 4^8,50^. Even though little information is known about the physiological and genetic mechanisms causing the mophead phenotype, the GWAS and segregation of the discovered SNP in the F_2_ population does support the hypothesis that the appearance of the mophead phenotype is a qualitative rather than a quantitative change in *H. macrophylla*, as proposed by Uemachi and Okumura^8^. An insertion of a long terminal repeat retrotransposon into the locus controlling inflorescence type was also proposed by the same research group through an observation of lacecap hydrangea cultivar mutation, but such a finding was not able to be connected to the current study due to limited genomic information. While genetic markers linked to inflorescence type in bigleaf hydrangea have been reported previously^46,50^, the single marker discovered and utilized herein has advantages for use in MAS. The previously published markers must be used in combination for marker-assisted selection while the SNP detailed here can be used alone. Also, SNPs may be converted into high-throughput marker systems for selection in very large populations. Further studies, such as high density genetic linkage map construction, whole-genome sequencing, and gene-expression analyses, are required to reveal the genes associated with this particular trait.

Twenty-three SNPs were detected to be associated with remontancy using GLM but none of them were detected with MLM. Possible reasons include, but are not limited to, small population size, lack of phenotypic variation, and the stringent requirements of MLM for qualitative traits^51^. However, the most likely reason is an incomplete understanding of remontancy combined with a history of poor phenotyping that has led to mixed reporting as to whether a cultivar is remontant, free-flowering, both, or neither. As an important trait in *H. macrophylla*, remontancy has been observed and studied for almost two decades^52^. However, little progress has been made to understand the genetic nature behind this trait. Segregation of remontant and non-remontant progenies in a recent developed breeding population indicated that remontancy was recessively inherited (unpublished data). In the current study, non-remontant hydrangeas were characterized by a single flush of inflorescences on old wood, while remontant hydrangeas flowered on both old wood and new growth. However, a third phenotype was also observed: some cultivars flowered continuously on old wood. For these cultivars, floral buds initiated the previous fall did not open in a single flush, but opened individually throughout the growing season. These cultivars may be mistakenly identified as remontant if phenotyping consists of counting new inflorescences throughout the growing season without removing previous season’s growth or noting where the flowering occurs (e.g.,^53,54^). Similar phenotypes were also observed in dwarf lilac where a non-complete and nonstable remontancy (semi-remontancy) was observed and a two-gene model was found to fit the observed phenotypic segregation ratio better than a one-gene model^55^. Segregation ratios that fit a two-gene model have not been tested in hydrangea given that semi-remontancy has not been treated as an individual phenotype. More rigorous phenotyping efforts are necessary for future studies in order to elucidate the genetic nature of remontancy in hydrangea.

Traditional breeding of hydrangeas for trait improvement takes at least two years before promising plants can be selected, cloned, and evaluated in large numbers. The time-to-market for new cultivars can be reduced greatly by identifying molecular markers associated with specific traits. In the present study, a SNP associated with inflorescence type was identified and converted to both CAPS and KASP-SNP markers for small and large population selection in *H.* *macrophylla*. Inflorescence type was detected with 100% accuracy using the CAPS marker in a validation panel. KASP-SNP genotyping revealed that 76% of progenies in an F_2_ breeding population produced lacecap inflorescences. These plants could be discarded as seedlings before further container or field evaluation in a breeding program targeting mophead inflorescence type. Furthermore, the SNP markers developed here will be useful to understand the biological mechanisms behind inflorescence architecture as improved genome resources for hydrangea become available.

## Conclusions

A large number of SNPs developed through GBS were used to perform GWAS to investigate the genetic control of inflorescence type and remontancy in *H*. *macrophylla*. Two statistical models identified a SNP locus that was tightly linked to inflorescence type of hydrangea. This particular SNP locus (*Hy_CAPS_Inflo*) was converted to a CAPS-SNP and KASP-SNP marker which will be useful for MAS in hydrangea breeding programs. Even though no SNP associated with remontancy was identified in two simulation models, insights have been provided to further the genetic study of remontancy in hydrangea.

## Supplementary information

Supplementary Information
