# Supplementary material for: Genome-wide association studies for inflorescence type and remontancy in Hydrangea macrophylla
Source: Hortic Res. 2020 Mar 1;7:27. doi: 10.1038/s41438-020-0255-y (PMC7049302; doi:10.1038/s41438-020-0255-y)
Supplement: Supplementary file 1 — Supplementary Information [file 41438_2020_255_MOESM1_ESM.docx]

Supplementary Table S1. Cultivars used for genome-wide association studies of inflorescence type and remontancy.

| ID | Name | Subspecies | Inflorescence* | Remontant |
| --- | --- | --- | --- | --- |
| 1 | All Summer Beauty | *macrophylla* | M | no |
| 2 | Ayesha (Uzu Azisai) | *macrophylla* | M | no |
| 3 | Beaute Vendomoise | *macrophylla* | L | no |
| 4 | Big Daddy | *macrophylla* | M | yes |
| 5 | Blauer Prinz | *macrophylla* | M | no |
| 6 | Blauling | *macrophylla* | L | na |
| 7 | Blaumeise | *macrophylla* | L | no |
| 9 | Blue Billow | *serrata* | L | no |
| 8 | Blue Danube | *macrophylla* | M | no |
| 10 | Blushing Bride | *macrophylla* | M | yes |
| 11 | Bridal Bouquet | *macrophylla* | M | no |
| 12 | David Ramsey | *macrophylla* | M | yes |
| 13 | Diadem | *serrata* | L | no |
| 14 | Domotoi | *macrophylla* | M | no |
| 15 | Dooley | *macrophylla* | M | yes |
| 16 | Double Dare | *macrophylla* | L | na |
| 17 | Elster | *macrophylla* | L | no |
| 18 | Endless Summer (Bailmer) | *macrophylla* | M | yes |
| 19 | Everlasting Amethyst | *macrophylla* | M | no |
| 20 | Everlasting Ocean | *macrophylla* | M | no |
| 21 | Everlasting Revolution | *macrophylla* | M | yes |
| 22 | Fasan | *macrophylla* | L | no |
| 23 | Floralia | *macrophylla* | M | no |
| 24 | Forever and Ever (Early Sensation) | *macrophylla* | M | yes |
| 25 | Fuji WaterFall | hybrid | L | no |
| 26 | General Vicomtesse de Vibraye | *macrophylla* | M | no |
| 27 | Glowing Embers | *macrophylla* | M | no |
| 28 | Grossman | *macrophylla* | M | na |
| 29 | Hamburg | *macrophylla* | M | no |
| 30 | Hanabi | hybrid | L | no |
| 31 | Heinrich Seidel | *macrophylla* | M | no |
| 32 | Holstein | *macrophylla* | M | no |
| 33 | Horben | *macrophylla* | M | no |
| 34 | Kern | *macrophylla* | M | na |
| 35 | Kiyosumi | *serrata* | L | no |
| 36 | Koby | *macrophylla* | L | no |
| 37 | Konigstein | *macrophylla* | M | no |
| 38 | LA Dreamin | *macrophylla* | M | yes |
| ID | Name | Subspecies | Inflorescence* | Remontant |
| 39 | Lady in Red | hybrid | L | no |
| 40 | Lemon Zest | *macrophylla* | M | no |
| 41 | Lemonhoff | *macrophylla* | L | no |
| 42 | Let’s Dance Moonlight (Robert) | *macrophylla* | M | yes |
| 43 | Libelle | *macrophylla* | L | no |
| 44 | Libelle White | *macrophylla* | L | no |
| 45 | Light of Day | *macrophylla* | L | no |
| 46 | Lilacina | *macrophylla* | L | yes |
| 47 | Little Geisha | *serrata* | M | no |
| 48 | Maculata | *macrophylla* | L | no |
| 49 | Madam Emile Mouillère | *macrophylla* | M | no |
| 50 | Mariesii Perfecta | *macrophylla* | L | no |
| 51 | Masja | *macrophylla* | M | no |
| 52 | Merritt's Supreme | *macrophylla* | M | no |
| 53 | Miss Hepburn | *macrophylla* | M | no |
| 54 | Monmar | *macrophylla* | M | yes |
| 55 | Mousseline | *macrophylla* | M | no |
| 56 | Mystical Everlasting Amethyst | *macrophylla* | M | no |
| 57 | Niedersachen | *macrophylla* | M | no |
| 58 | Nigra | *macrophylla* | M | no |
| 59 | Nikko Blue | *macrophylla* | M | yes |
| 60 | Oregon Pride | *macrophylla* | M | no |
| 62 | Penny Mac | *macrophylla* | M | yes |
| 61 | Pia | *macrophylla* | M | no |
| 63 | Pretty Maiden | *serrata* | L | no |
| 64 | Preziosa | hybrid | M | no |
| 65 | Princess Juliana | *macrophylla* | M | no |
| 66 | Regula | *macrophylla* | M | no |
| 67 | Rhonda | *macrophylla* | M | na |
| 68 | Sabrina | *macrophylla* | L | no |
| 69 | Sadie Ray | *macrophylla* | M | no |
| 70 | Sharona | *macrophylla* | M | no |
| 71 | Sol | *macrophylla* | L | no |
| 72 | Souvenir du President Doumer | *macrophylla* | M | no |
| 73 | Stella | *macrophylla* | M | no |
| 74 | Sybilla | *macrophylla* | M | no |
| 75 | Taube | *macrophylla* | L | no |
| 76 | Todi | *macrophylla* | M | no |
| 77 | Trophee | *macrophylla* | M | no |
| ID | Name | Subspecies | Inflorescence* | Remontant |
| 78 | VanHoose White | *macrophylla* | L | na |
| 79 | Veitchii | *macrophylla* | L | no |
| 80 | Wayne's White | *macrophylla* | L | no |
| 81 | Weidler's Blue | *macrophylla* | L | no |
| 82 | White Wave | *macrophylla* | L | no |
| 83 | Woodlander | *serrata* | L | no |
| Inflorescence*: M, mophead; L, lacecap. | | | | |
| na: not avaliable | | | | |

Supplementary Table S2. Cultivars and breeding materials used for Cleaved Amplified Polymorphic Sequences (CAPS) marker validation.

| **Lane** | **Name** | **Inflorescence** | **Source** |
| --- | --- | --- | --- |
| 1 | Endless Summer | M | Cultivar |
| 2 | 1150-02 | M | Breeding material |
| 3 | 1146-10 | M | Breeding material |
| 4 | 1146-14 | M | Breeding material |
| 5 | 0875-165 | M | Breeding material |
| 6 | 1228-074 | M | Breeding material |
| 7 | Uzu Azisai | M | Cultivar |
| 8 | Kluis Superba | M | Cultivar |
| 9 | Mini Penny | M | Cultivar |
| 10 | Veitchii | L | Cultivar |
| 11 | 0872-075 | L | Breeding material |
| 12 | 0872-182 | L | Breeding material |
| 13 | 1203-02 | L | Breeding material |
| 14 | 1203-22 | L | Breeding material |
| 15 | Miyama Yae Murasaki | L | Cultivar |
| 16 | Kurohime | L | Cultivar |
| 17 | 0872-053 | L | Breeding material |
| 18 | 0872-076 | L | Breeding material |
